# Characterizing the Helminth Community of the Mountain Gazelle (*Gazella gazella* Pallas, 1766) Through DNA Metabarcoding

**DOI:** 10.1007/s11686-025-01018-x

**Published:** 2025-04-03

**Authors:** Mina Cansu Karaer, Büşra Karataş, Elif Madak, Hande İrem Sönmez, Emre Keskin, Hıfsı Oğuz Sarımehmetoğlu, Tolga Kankılıç, Çağatay Tavşanoğlu

**Affiliations:** 1 Institute of Science, Hacettepe University, Ankara, Türkiye; 2 Division of Ecology, Department of Biology, Hacettepe University, Ankara, Türkiye; 3 Food and Agriculture Vocational School, Çankırı Karatekin University, Çankırı, Türkiye; 4 Institute of Preclinical Sciences, Veterinary Faculty, University of Ljubljana, Ljubljana, Slovenia; 5 Evolutionary Genetics Laboratory (eGL), Department of Fisheries and Aquaculture, Agricultural Faculty, Ankara University, Ankara, Türkiye; 6 AgriGenomics Hub Animal and Plant Genomics Research Innovation Centre, Ankara, Türkiye; 7 Graduate School of Natural and Applied Sciences Department of Biology, Ankara University, Ankara, Türkiye; 8 Institute of Health Sciences, Department of Parasitology, Ankara University, Ankara, Türkiye; 9 Department of Parasitology, Faculty of Veterinary Medicine, Ankara University, Ankara, Türkiye; 10 Aquaculture Research and Application Center (ASAUM), Ankara University, Ankara, Türkiye; 11 Department of Biology, Sabire Yazıcı Faculty of Science and Letter, Aksaray University, Aksaray, Türkiye

**Keywords:** Amplicon sequencing, *Gazella gazella*, Helminths, Metabarcoding, Parasite identification

## Abstract

**Purpose:**

Understanding parasite diversity in wild and captive animal populations is vital for their individual health and ecosystem dynamics. The helminth community in mountain gazelles (*Gazella gazella* Pallas, 1766), particularly in the isolated northernmost population in Türkiye, remains poorly understood, posing challenges for conservation. This study aimed to identify and compare the diversity of helminths in captive and free-ranging mountain gazelles in Hatay, Türkiye, while assessing potential zoonotic risks.

**Methods:**

We collected a total of 188 fresh fecal samples from both captive and free-ranging populations. The samples were analyzed using DNA metabarcoding to assess helminth species and their species diversity across seasons.

**Results:**

Our findings revealed eight helminth taxa in *Gazella gazella*, including six intestinal and two lung nematodes, with four of these species previously unreported in Türkiye. We also found seasonal differences in helminth composition and abundance.

**Conclusions:**

The identification of these helminth taxa highlights the value of advanced molecular techniques in uncovering parasite diversity in ungulates. Seasonal differences in helminth composition and abundance, and the biological characteristics of the detected helminth species align with the climatic parameters of the seasons in which they were identified.

## Introduction

Wildlife is the primary source of most infectious diseases, which create a risk to human and livestock health [1]. Parasitic infections are among the diseases that can spread across animals, livestock, and human populations through direct or indirect contact. Additionally, they may have negative impacts on the human economy by affecting food security due to livestock-related consequences, which could result in a loss of profits [1, 2]. Parasite sharing can also arise from ecological interactions including mutualism, competition, and predation. Among the most well-studied direct ecological interactions are those between predators and prey [3]. These interactions can open up significant paths for parasites to spread to new hosts, including direct contact with skin, and bodily fluids as well as ingestion of the parasite stages (larvae, eggs or adults). Therefore, it’s possible that the parasites present in some carnivore species were transferred by their ungulate prey [3]. Different kinds of parasites, some of which may be lethal depending on the parasite’s species or load, infect wild ruminants [4, 5]. Most of the time, common grazing pastures are shared by domestic and wild ruminants. Therefore, there is an extremely likely chance that parasite infections would spread from wild ruminants to domestic animals and vice versa [6] through contaminated food, plants, soil, or other materials.

The literature places particular emphasis on the presence or absence of parasites and the parasite load of wild herbivores [7–9]. The fact that parasites affect ungulates, like other animals, in terms of growth and development, immune systems, reproduction, behavioral patterns, and survival is one of the main causes of this [8, 10]. Moreover, research has shown that the abundance of parasites can also influence the distribution of individuals [10]. Wildlife infections are known to endanger both people and domestic animals who share an environment with them. Consequently, comprehending the host-parasite dynamics become crucial for the sustainable management and protection of species [11]. Furthermore, parasite disease outbreaks in livestock might result in decreased output and affect the economy.

Different laboratory techniques have been employed to determine the prevalence of endoparasites [1, 12, 13]. To date, conventional methods has been predominantly performed to assess endoparasites of wild ungulates [1, 14]. Recently, metabarcoding has started to be used especially in the detection of endoparasites [15–18]. This method has been also using to determine endoparasites in wild ungulates including *Bison bison* [19], *Rangifer tarandus* [20], *Alces alces* [21] and in some studies in several species [9, 22–24]. However, in *Gazella* species or phylogenetically close species, molecular approach has rarely been used in the identification of endoparasites *(G. dorcas*, *G. leptoceros*: [25]; *Nanger dama*: [26]; *Procapra przewalskii*: [27] and this research area has mainly based on conventional methods (*Gazella cuvieri*, *G. dama*, and *G. dorcas*: [28]; *Gazella grantii*: [29]; *Gazella gazella farasani*: [30]; *Gazella gazella farasani*: [31]; *Gazella subgutturosa*: [32]; *Gazella gazella*: [33, 34]; *Gazella cuvieri*: [35]).

Before the advent of NGS methods, various genetic and molecular techniques could be employed for the diagnosis of parasites. Polymerase Chain Reaction (PCR) is commonly used to amplify specific DNA or RNA sequences, allowing for the detection of parasitic presence [36]. Reverse Transcription PCR (RT-PCR) is particularly useful for identifying parasites [37]. Restriction Fragment Length Polymorphism (RFLP) helps assess genetic diversity by analyzing the sizes of DNA fragments produced by specific enzymes [38]. Traditional Sanger sequencing is employed for determining specific gene sequences, while hybridization techniques, such as Southern and Northern blotting, facilitate the identification of target DNA or RNA [39]. Additionally, microsatellite analysis is used to evaluate genetic diversity among parasites [40]. Methods such as PCR, RT-PCR, RFLP, Sanger sequencing, and microsatellite analysis have various limitations, including sensitivity, specificity, time, cost, and limited information [41, 42]. Next-generation sequencing (NGS) has swiftly produced substantial datasets from parasitic species derived from a single individual, population, or environmental sample in a single run, addressing the constraints of cutting-edge molecular techniques. Furthermore, NGS technology has several benefits over standard Sanger sequencing, such as high throughput, lower costs, faster processing, and great sensitivity [43].

Determining the species involved in the ecological processes is necessary for further ecological investigations. Obtaining such biodiversity data for plants and animals using morphological traits to identify field-collected samples necessitates a substantial sampling effort in addition to a variety of taxonomic knowledge that is rarely found within a single scientific group. This identification process has been made much simpler by the recent discovery of DNA-based techniques for species identification, or DNA barcoding [44]. The technique which provides us with the identification of several taxa present in a single environmental sample is the main objective of DNA metabarcoding [45].

Due to their frequent interaction with wild ungulates that are free to roam, people of rural areas are more likely to contract the disease. Usually, the primary source of income in this region is animal husbandry, therefore parasite illnesses can also have an indirect impact on these populations. On the other hand, nothing is still known about the parasite fauna of the local species except two gastrointestinal parasites studies which were conducted on the mountain gazelle (*Gazella gazella* Pallas, 1766) [33, 34]. This work intends to close this gap by using DNA metabarcoding to evaluate the overall gastrointestinal helminths in captive and free-ranging mountain gazelle populations in Hatay, Türkiye throughout a year. This information will offer crucial baseline data that will support upcoming surveillance initiatives. Examining the gastrointestinal helminths of the mountain gazelle populations is important for the survival and population growth of the species itself, and it should be a top priority for conservation efforts given the coexistence of other animal species that share food and habitat in the region. By investigating the helminth diversity, we also aimed to reveal the zoonotic transmission potential of identified helminths.

## Materials and Methods

### The Study Species

The genus *Gazella* is a highly diverse group within the family Bovidae, order Artiodactyla, and includes several endangered species. Although they can also be found on the Indian subcontinent, southwest and central Asia, and the deserts, grasslands, and savannas of Africa are the primary habitats for gazelles [46]. *Gazella gazella* (the mountain gazelle) is one of the endangered species of the genus according to the IUCN Red List [47]. The species was previously widespread across the Mediterranean coasts of the Middle East, including Israel, Palestine, Jordan, Türkiye, Syria, Lebanon, and even Sinai, Egypt. Nowadays, the majority of the current population of *G. gazella* is concentrated in Israel (approx. 5000 individuals [48]), and Türkiye (approx. 1500 individuals, Karaer et al. unpublished).

### The Study Site

The study was conducted in the Hatay Mountain Gazelle Wildlife Development Area, encompassing 13,288 ha area, near the Türkiye-Syria border (36°32’ N, 36°32’ E; 200–450 m). This site is the only and primary range of the northernmost population of *Gazella gazella* [49]. The area is primarily composed of grassland vegetation with a few patches of shrubland, large expanses of cropland, and rocky hills. The habitat is actively used by mountain gazelles all year round. Additionally, captive mountain gazelles are also present at the Hatay Mountain Gazelle Production Centre, located within the Wildlife Development Area.

The study site is home to a diverse array of mammal species including some carnivores. *Canis lupus* (gray wolf), and *Vulpes vulpes* (red fox) are considered natural predators of mountain gazelles, while other species include *Hyaena hyaena* (striped hyena), *Felis chaus* (jungle cat), *Felis silvestris* (wildcat), *Hystrix indica* (Indian porcupine), *Lepus europaeus* (wild rabbit), and *Meles meles* (European badger). Year-round observations of domestic sheep herds are also common in the research area [50, 51].

To enable the ecological assessment of the obtained results, the climate conditions of the sampling area were considered during each sampling period. The climate conditions in Hatay during the sampling periods, based on data from the Turkish State Meteorological Service, varied significantly. In December 2022, the average temperature was 11.9 °C, with an average humidity of 72.4% and total precipitation of 42.2 mm. By April 2023, temperatures rose to an average of 18.5 °C, accompanied by a decrease in humidity to 54.3% and total precipitation of 21.6 mm. In July 2023, the temperature peaked at 32.8 °C, with humidity dropping further to 38.7% and no recorded precipitation. Finally, in September 2023, the average temperature was 28.7 °C, humidity was at 50.1%, and total precipitation was 0.6 mm.

### Fecal Sampling and DNA Isolation

Field sampling was conducted between December 2022 and September 2023 in the Hatay Mountain Gazelle Wildlife Development Area. Fresh fecal samples were collected from captive and free-ranging mountain gazelles based on a standardized observation protocol. Individuals were initially spotted using binoculars from a distance of at least 500 m, allowing us to quickly reach feces by car or on foot immediately after defecation. Since the fresh fecal samples were collected right after defecation and from different locations, each sample from a single sampling period was highly likely to belong to a distinct individual.

A total of 188 fecal samples were collected during four sampling periods: 43 samples in December 2022 (34 free-ranging, 9 captive), 48 samples in April 2023 (40 free-ranging, 8 captive), 48 samples in July 2023 (39 free-ranging, 9 captive), and 49 samples in September 2023 (38 free-ranging, 11 captive). Right after collection, about 3 g of feces from every sample was put in sterile plastic tubes and kept under refrigeration during transport to the laboratory.

Upon arrival at the laboratory, the samples were stored at − 20 °C until DNA extraction was done. DNA was extracted from the samples using the GeneMatrix Bio-Trace DNA Purification Kit (EURx, Poland) according to the manufacturer’s instructions with minor modifications to optimize yield from fecal specimens.

### DNA Amplifications for DNA Metabarcoding

DNA amplifications were carried out in a final volume of 50 µL, using 1 µL of diluted DNA extract as a template. The PCR was conducted using 18 S primers, PCR forward primer (5′- GGCCGTTCTTAGTTGGTGGA − 3′) and PCR reverse primers (5′- CCCGGACATCTAAGGGCATC − 3′) [52]. All the PCR reactions were performed with Q5^®^ High-Fidelity DNA Polymerase (NEB). A total of 10 µL first PCR MasterMix consisted of 5 µL of 2× Hot Start Master Mix, 0,5 µL of forward and reverse primers (5 pmol/µL), and 1 µL DNA and 3 µL water. The amplification was conducted in accordance with the following protocol: an initial denaturation at 98 °C for 30 s, followed by 30 cycles of denaturation at 98 °C for 10 s, primer annealing at 60 °C for 30 s, and extension at 72 °C for 30 s, with final elongation at 72 °C for 2 min, and cooled down at 4 °C after the PCR procedure, amplicons were stored at -20 °C.

From December 2022, 30 samples (26 samples from free-ranging population, four samples from captive population), from April 2023 44 samples (38 samples from free-ranging population, six samples from captive population), from July 2023 29 samples (27 samples from free-ranging population, two samples from captive population), and from September 2023 17 samples (14 samples from free-ranging population, three samples from captive population) samples were showed visible bands.

### DNA Library Preparation and Quality Assessment

The NEBNext^®^ Ultra™ II DNA Library Prep Kit for Illumina (New England Biolabs) was used to prepare the library. For library preparation, the same kit’s cleaning protocol was used to purify the PCR products and add adapters. These adapters enable the DNA fragments to attach to the Illumina NovaSeq™ 6000 device for sequencing. Subsequently, an indexing step was performed where barcodes (short nucleotide sequences) were added in different combinations for each sample, allowing for differentiation of the samples after sequencing. In the final stage, the prepared libraries were cleaned to remove excess primers and small DNA fragments. All these procedures were conducted using the NEBNext^®^ Ultra™ II DNA Library Prep Kit for Illumina (New England Biolabs), following the protocol specified on the manufacturer’s website. Library quality control was performed using the Agilent 2100 Bioanalyzer (Agilent, Santa Clara, USA) with the Agilent High Sensitivity DNA Kit (Agilent, Palo Alto, USA) and the ThermoFisher Invitrogen Qubit Flex Fluorometer (ThermoFisher, Waltham, Massachusetts, USA) with the Vazyme 1x dsDNA High-Sensitivity Assay (Vazyme, Nanjing, China) to measure concentration and size distribution. Libraries passing quality control were normalized to the concentrations required for sequencing, ensuring that each library was equally represented in a single tube. The normalized library was then loaded onto the Novaseq 6000 S2 flow cell. Sequencing was initiated using the Illumina NovaSeq™ 6000 System, which read the DNA sequences, collected the data, and prepared it for analysis.

### Bioinformatics and Statistical Analysis

Bioinformatics analyses were completed through the Linux/Unix-based operating system terminal. The quality controls of the “.fastq” formatted forward and reverse read sequences obtained from the Illumina NovaSeq™ 6000 device was checked with the FASTQC program. Further analyses were performed using the “ObiTools” package. Sequences were aligned and merged with the code “illuminapairedend” with phred score threshold of ≥ 30. After that, filtering of non-merged reads (obigrep), trimming forward and reverse primers at both ends by allowing a maximum of 3 mismatches (tagcleaner), cleaning of duplicate data (obiuniq), and cleaning of unnecessary data from each sample header (obiannotate) were performed respectively. The expression ‘uniq’ was used in all sequence data obtained as a result of this workflow. All these files were matched with the online NCBI GenBank database using megablast tool of Geneious Prime.

Helminth abundance was quantified by tallying the sequence reads mapping to each helminth taxon in every PCR-positive sample. The raw read counts, which were derived following quality filtering, primer trimming, and duplicate removal, constituted a semi-quantitative estimate of the helminth burden. Abundance comparisons were restricted to samples with detectable helminth sequences. The read counts per taxon were then used in generalized linear models with a Poisson distribution to test the impact of seasonality and captivity on helminth abundance.

Analyses were performed using R (version 3.6 [53])., We compared the presence or absence of gastrointestinal parasite species between captive and free-ranging gazelle populations, considering captivity and seasons. Since the abundance data of gastrointestinal parasites had excess zeros, we used a two-staged modelling approaches to deal with this problem. Thus, to reveal the effect of season and captivity status on the presence and abundance of gastrointestinal parasites, we used generalized linear models assuming both binomial (using presence and absence data) and Poisson distributions (using abundance data where only fecal samples with endoparasite records included). To further inspect the differences among seasons, we made multiple comparisons following generalized linear model analyses by estimating marginal means based on Tukey HSD adjustment method by using the emmeans package [54].

## Results

Among 188 fresh fecal samples we collected from the field, only 120 samples contained visible PCR bands and were further processed to identify helminths using DNA metabarcoding. The other 68 samples lacked PCR bands, probably because of poor quality of DNA or the existence of inhibitors, and were therefore left out of further analysis. Of the 120 PCR-positive samples, 63 were found to have matches in the MegaBLAST results, 49 of which were deemed helminth-specific. There is, therefore, a 40.8% prevalence of helminths in the PCR-positive samples. If the 68 PCR-negative samples are removed from the helminth population, then the prevalence in the 188 samples would be 26.1%.

We identified a total of six different gastrointestinal nematodes and two lungworm species from these 49 samples. Positive results obtained in the samples varied among season samplings. Specifically, we found evidence for helminths in 12 fecal samples in December (one in captive, 11 in free-ranging population), in 20 samples in April (six in captive, 14 in free-ranging population), in 12 samples in July (all in free-ranging population), five samples in September (all in free-ranging population).

Based on comparisons with the GenBank database, the most frequently encountered nematodes were *Trichostrongylus colubriformis* (read count: 271) and *Umingmakstrongylus pallikuukensis* (read count: 258). Following these species, in order, *Protostrongylus rupicaprae* (read count: 61), *Nematodirus spathiger* (read count: 18), *Haemonchus contortus* (read count: 14), *Skrjabinema kamosika* (read count: 9), *Trichuris skrjabini* (read count: 4), and *Chabaudstrongylus ninhae* (read count: 3) were also encountered. The sequencing results reveal intriguing insights into nematode prevalence in fecal samples, highlighting significant differences between captive and free-ranging populations (Table 1). *Umingmakstrongylus pallikuukensis* topped the list, detected in a remarkable 83.67% of samples (Captive: 5/7, Free-ranging: 36/42). Close behind, *Trichostrongylus colubriformis* showed an impressive detection rate of 81.63% (Captive: 6/7, Free-ranging: 34/42). *Protostrongylus rupicaprae* was present in 40.81% of the samples (Captive: 3/7, Free-ranging: 17/42), while *Nematodirus spathiger* appeared in 20.40% (Captive: 1/7, Free-ranging: 9/42). *Haemonchus contortus* followed, detected in 16.32% of the samples (Captive: 1/7, Free-ranging: 7/42). *Skrjabinema kamosika* was found in 14.28% of the samples (Captive: 0/7, Free-ranging: 8/42), and *Chabaudstrongylus ninhae* made a minor appearance at 4.08% (Captive: 0/7, Free-ranging: 2/42). Finally, *Trichuris skrjabini* was detected in just 2.04% of the samples (Captive: 0/7, Free-ranging: 1/42). These findings underscore the varied nematode landscape across different environments, revealing notable patterns of parasitic occurrence.

**Table 1:** Average values of relative abundance of nematodes found in fecal samples based on captivity and seasonal conditions. Taxa marked with an “†” sign are identified as *C. ninhae*, *S. kamosika*, *U. pallikuukensis*, and *P. rupicaprae*, respectively. These taxa have not been previously recorded in Türkiye and close regions (except *P*. *rupicaprae* which found in Italy) and due to the lack of morphological verification, they are reported at the genus level to avoid false positive results. The abbreviation “C” denotes the captive population, while “FR” indicates the free-ranging population

	Dec 22		Apr 23		Jul 23		Sep 23	
Taxon	C	FR	C	FR	C	FR	C	FR
**Gastrointestinal nematodes**								
*Chabaudstrongylus* spp. †	0	0.04	0	0.05	0	0	0	0
*Haemonchus contortus*	0	0.31	0.17	0.13	0	0	0	0
*Nematodirus spathiger*	0	0.23	0.50	0.24	0	0	0	0
*Skrjabinema* spp. †	0	0.12	0.00	0.13	0	0.04	0	0
*Trichostrongylus colubriformis*	0.25	5.54	2.50	1.37	0	1.52	0	1.29
*Trichuris skrjabini*	0	0	0	0	0	0.15	0	0
**Lungworms**								
*Umingmakstrongylus* spp. †	0.25	1.77	1.17	1.37	0	4.26	0	2.64
*Protostrongylus* spp. †	0.25	1.15	0.50	0.29	0	0.15	0	0.86

Presence-absence and abundance analyses of each nematode species were conducted on the detected fecal samples (Table 2). Significant differences were found between December-September and September-April (*P* < 0.05) when evaluating the dataset. No significant differences were observed in other seasons (*P* > 0.05). It was found that the presence of parasites in September was statistically lower compared to April and December (*P* < 0.005). Additionally, parasite abundance in April was statistically significantly lower compared to December, July, and September (*P* < 0.0001). In the free-ranging population, although no statistically significant seasonal difference was found in the presence-absence analysis (*P* > 0.05), seasonal differences were detected in the abundance analysis. It was found that abundance values in April were significantly lower compared to other seasons (*P* < 0.001). In the captive population, no statistically significant difference was observed in the abundance analysis (*P* > 0.05). However, the possibility that this result might be due to the small sample size in the captive population cannot be ruled out.

**Table 2:** Summary of the generalized linear models (deviation analysis) based on the binomial distribution regarding the effects of season and captivity status on parasite presence and abundance in feces. Presence and abundance analyses were performed via GLMs assuming binomial and Poisson distributions. In the abundance analysis, only fecal samples in which parasites were detected were considered

Factor	d.f.	Deviance	Explained deviance (%)	*P*
Presence				
Null	-	761.4	-	-
Season	3	12.7	1.7	0.0005
Captivity	1	~ 0	0.0	> 0.05
Season × Captivity	7	25.5	3.3	0.0006
Abundance				
Null	-	855.8	-	-
Season	3	98.2	11.5	< 0.0001
Captivity	1	39.4	4.6	< 0.0001
Season × Captivity	5	121.8	14.2	< 0.0001

## Discussion

Our findings revealed the presence of eight helminth taxa, including six intestinal nematodes and two lung nematodes, in the mountain gazelle population in Hatay province, Türkiye. Among these taxa, four were previously unreported in Türkiye. These results provide significant insights for the conservation of endangered mountain gazelles, which are distributed in a restricted area within the study region. The findings are also relevant considering the potential for other wild animals or domesticated ruminants in the region to serve as reservoirs for parasite infections as previous studies conducted both globally and within Türkiye have shown that wild ruminants commonly transmit infections to domesticated ruminants or vice versa [4, 55].

The metabarcoding analysis uncovered notable seasonal trends in helminth diversity, emphasizing the impact of environmental factors on parasite populations. The occurrence of *Trichostrongylus colubriformis* and *Haemonchus contortus* corresponds with fluctuations in rainfall and temperature, highlighting the influence of climatic conditions on parasite behaviour. The ability to correlate genetic data with ecological parameters is a notable advantage of metabarcoding, facilitating a more profound comprehension of host-parasite interactions over various temporal and spatial dimensions.

In our study, we employed molecular-based approach to assesses the diversity of helminths. In previous studies on mountain gazelle helminths that used conventional methods [33, 34], the main gastrointestinal nematodes identified were *Nematodirus* spp., *Trichuris* spp., and *Marshallagia* spp [34]., and *Nematodirus* spp., *Marshallagia* spp., and *Trichostrongylus* spp [33]. No other studies have been conducted on gastrointestinal helminths of *Gazella gazella* aside from these two. Both studies used conventional methods for gastrointestinal helminth diagnosis, so the identified helminths were limited to the genus level. This study illustrates the advantages of DNA metabarcoding compared to traditional methods in detailing the helminth diversity found in *Gazella gazella*. Although the morphological identification of helminths and other pathogens is indispensable to identify these organisms, these traditional techniques also have their own limitations due to the degradation of parasite stages in fecal samples. In addition to these traditional morphological identification techniques, using metabarcoding facilitates species-level identification with enhanced sensitivity and throughput. In our study, for instance, this molecular approach revealed taxa, such as *Chabaudstrongylus ninhae* and *Skrjabinema kamosika*, that had not been previously documented in Türkiye, highlighting its ability to unveil hidden biodiversity and deepen our comprehension of parasite ecology in wildlife populations.

*Nematodirus* spp., although more commonly observed in young individuals during spring, also poses a risk of infection for adult populations [56]. According to our findings, *N. spathiger* is most frequently observed in April and has not been detected outside of the December to April period. Due to the high temperatures in the study area, *N. spathiger* may not have been observed in our samples from the summer sampling period. It is also known that *N. spathiger* is present in domestic ruminants in Türkiye [57]. Furthermore, previous studies on *Gazella* species (*Gazella subgutturosa*: [58]; *G. cuvieri*, *G. dama*, and *G. dorcas*: [59]; *Gazella subgutturosa marica* and *Gazella gazella*: [60]; *Gazella cuvieri* and *Gazella dorcas*: [61]; *Gazella dorcas* and *Gazella leptoceros*: [25]; *Gazella subgutturosa*: [62]; *Gazella dorcas*: [63]; *Gazella dorcas*: [64]) frequently reported this species.

The *Trichostrongylus* genus (Nematoda: Trichostrongylidae) is a zoonotic nematode with a broad geographic distribution. Humans can become infected with *Trichostrongylus* species through the ingestion of third-stage larvae, which are released from the eggs present in the feces of infected livestock, both domestic and wild [22]. These larvae can contaminate the environment, including water sources, soil and vegetables, particularly in areas where animal feces are used as fertilizer or where poor sanitation practices exist [6]. Once ingested, the larvae can migrate through the human gastrointestinal system, potentially leading to infection. In Türkiye, *T. colubriformis* has been detected in both wild and domestic animals [65, 66]. In our study, *T. colubriformis* was identified as a nematode present across all seasons. In our study area, *T. colubriformis* was most frequently observed in April and December. The decline in average abundance observed in July and September can be attributed to the inability of the L1 and L2 stages, which are sensitive to drought, to develop into the infective L3 stage (Table 1), likely due to higher temperatures during these periods. Previous studies on *Gazella* species (*Gazella subgutturosa*: [58]; *Gazella dorcas*: [67]) have also reported the presence of *Trichostrongylus colubriformis*.

The eggs and larvae of *Haemonchus* spp. are not resistant to cold and drought, with optimal conditions for larval development occurring during mild, rainy winters [68]. In regions with Mediterranean climates, the development of infective larvae of *H. contortus* is generally restricted to specific short periods of the year, particularly in autumn and spring, when sufficient warmth and rainfall coincide [69]. In areas with especially dry summers, *H. contortus* is rarely detected or may not appear at all. In our study, *H. contortus* was identified in December and April. This finding aligns with the known epidemiology of the species, considering the average rainfall and temperature values of the study area. Studies conducted on *H. contortus* have reported its presence in many *Gazella* species (*Gazella bennettii*: [70]; *Gazella marica*: [71]) and other members of the Antilopini subfamily (*Eudorcas thomsonii*: [72]; *Eudorcas rufifrons*: [73]).

One of the most important factors in the development of *Trichuris* species is the high resistance of parasite eggs to climatic and environmental conditions [74]. The optimal temperature for maintaining egg viability and larval development of *Trichuris skrjabini* is 25 °C, while temperatures rising to 30 °C gradually reduce egg viability during embryogenesis. At low temperatures, metabolic processes during embryonic development slow down, causing larval development to halt and extending the developmental period within the egg [75]. Consequently, the observation of *T. skrjabini* only in July in our study reflects current environmental conditions and temperature, confirming its epidemiological relevance. Species-level detections of this nematode have previously been reported in other gazelle species (*Gazella bennettii*: [76]; *Gazella subgutturosa*: [77]).

Species of the genus *Protostrongylus* typically inhabit the lungs of ruminants and are widely distributed in mountainous areas with temperate, tropical, and subtropical climates [78]. In our study, *P. rupicaprae* was detected in every sampling period, indicating a high likelihood of interactions between definitive and intermediate hosts in the study area. This finding confirms the epidemiological relevance of the parasite, as it can complete its life cycle within these months. However, *P. rupicaprae* has not been previously reported in Türkiye. Therefore, the matching sequences were re-examined and analyzed for the possibility of other species. Among these, a 100% match was found with *P. rufescens*, a species previously reported in Türkiye. Given the high degree of similarity between the sequences of both species, as well as similar issues observed in other species discussed later, it is important to note that the primer used in the diagnosis of gastrointestinal parasites may lack specificity. This study has resulted in the identification of helminths that have not been previously catalogued in Türkiye, highlighting the significance of adding local biodiversity data to global repositories like NCBI. Through this approach, metabarcoding facilitates the development of databases tailored to specific regions, which are essential for tracking zoonotic risks and guiding conservation efforts. The high-resolution taxonomic data produced by this method can inform policies aimed at managing wildlife health and reducing the risks of cross-species transmission. Using additional primers in future studies could yield more precise results and allow for more accurate interpretations.

*Umingmakstrongylus pallikuukensis* is a lungworm that uses gastropods as intermediate hosts in its life cycle. The species identified through metabarcoding belongs to a group of parasitic nematodes in the family Protostrongylidae. *Umingmakstrongylus pallikuukensis* is generally found in Arctic regions [79–81], and there are no prior records of this species in Türkiye. However, this parasite was detected in December, April, July, and September (Table 1). To verify these findings, additional analyses were conducted, and other species with high sequence matching were identified. These included *Oslerus rostratus* (feline lungworm: [82]), *Crenosoma vulpis* (lungworm: [83]), *Metastrongylus pudendotectus* (lungworm: [84]), *Molineus patens* (gastrointestinal nematode: [85]), and *Prestwoodia delicata* (gastrointestinal nematode: [86]). None of the matching species, except for the identified one, have been reported in ruminants to date. The recovered sequences, which exhibited the most similar alignment to *U. pallikuukensis* in our reference dataset, can be attributed to the conserved nature of the targeted genetic region’s characteristics and hence ambiguous classification between related lungworm taxa. It is thus plausible that these sequences are from a misidentified lungworm species since the used marker was not adequate or could represent a closely related but undescribed species parasitizing *Gazella gazella*. Additional research involving other genetic markers coupled with more morphological studies will be significant in determining the appropriate taxonomic status of these lungworms. To obtain more accurate identification, further data collection and testing with different primers are required.

*Skrjabinema* spp. are nematodes that inhabit the large intestines of ruminants. Due to their direct life cycle, *Skrjabinema* spp. can potentially being observed throughout the year. In free-ranging animals, the primary route of infection is the ingestion of infective eggs from contaminated environments, such as water and pasture. Our study found a match with *S. kamosika*, however, according to the literature, this species has been identified only in the endemic Japanese species *Capricornis crispus*, using the 18 S rDNA primer [87]. This study also reported that *S. kamosika* is common in Japan and highlighted the complex evolutionary history of this parasite with other species in the same genus. It closely resembles *S. africana* and *S. alata* which are found in Africa. In Türkiye, the species *S. ovis* [88] is present, and previous studies have detected *S. ovis* in *Gazella* species (*Gazella subgutturosa*: [89]; *Gazella subgutturosa*: [77]; *Gazella subgutturosa*: [90]). Aside from these studies, *Skrjabinema* at the genus level has also been identified in *Gazella* species (*Gazella gazella*: [34]). We believe that the species identified in our study is likely *Skrjabinema ovis*. The species was detected in fecal samples collected in April and July, with low average abundance. Given the limited studies on *Skrjabinema* in *Gazella* species, we can also infer, based on our data, that *S. ovis* is not commonly found in these populations. On the other hand, since *S. kamosika* had not been previously reported in Türkiye, we repeated our analyses to avoid speculation. The repeated analyses still confirmed the presence of only *S. kamosika* with 100% match. More research and data are necessary to reach a more definitive conclusion.

*Chabaudstrongylus ninhae* is a trichostrongylid nematode that resides in the small intestine. Currently, there is currently no information available on the epidemiology of this parasite. To date, only two studies have been conducted on this species. The first study identified *C. ninhae* in *Muntiacus reevesi* (Chinese muntjac) on Izu-Oshima Island, Japan [91]. Fecal samples were collected in January (average temperature 9.4 °C, 2015), July (average temperature 26.1 °C, 2015), and October (average temperature 20.9 °C, 2016), and the presence of the species was detected in each research period. The frequency of the species in feces was highest in July, followed by October and January. The second study detected *C. ninhae* in *Cervus nippon centralis* (Sika deer) [92], using organs collected post-hunting from 2014 to 2019. This study suggested that *C. ninhae* is specific to the exotic Chinese muntjac and was introduced to Japan through this host. Comprehensive epidemiological research and close monitoring of *C. ninhae* distribution is therefore essential. In our study, *C. ninhae* was detected in December and April. Repeated analyses revealed high sequence matches with other species, including *Viannaia viannai*, *Travassostrongylus* spp., *Viannaia didelphis*, *Travassostrongylus callis*, *Viannaia hamata*, and *Viannaia minispicula*. All these species, along with *C. ninhae*, belong to the family Trichostrongylidae. The primers used in this study did not provide species-specific discrimination for this match.

The larger sample size of the free-ranging population compared to the captive population allowed statistically more precise and reliable results. In the captive population, the most frequently identified helminths were *T. colubriformis*, *U. pallikuukensis*, and *P. rupicaprae*, respectively. Along with these three species, *N. spathiger* and *H. contortus* were also found in the captive population. In the free-ranging population, all eight identified species were present, with *U. pallikuukensis*, *T. colubriformis*, and *P. rupicaprae* being the most frequently detected. The difference in the number of helminth species observed between the free-ranging and captive populations may be primarily due to the larger sample size of population size of the free-ranging group (> 1000 individuals) as well as its extensive habitat (> 10,000 hectares). The higher sampling intensity in the free-ranging population likely contributed to increased detection of species richness. Furthermore, the potential exposure to other wild and domestic animals may have allowed for a broader range of parasite species in free-ranging population. However, it is essential to interpret these findings carefully, as differences in sample size between the populations could be a significant contributing factor, if not the primary factor, in detecting greater species richness.

The metabarcoding approach has certain limitations, including the absence of truly universal primers, potential contamination issues, and challenges in accurately quantifying relative abundances. While molecular methods facilitate species-level identification of various life stages based on few morphological characters, they do not provide insight into whether the helminths are viable, infective, or capable of sustaining their life cycles. Compared to conventional methods, which rely on morphological identification of adult helminths, larvae, and eggs, molecular methods using fecal samples offer higher throughput, repeatability, and species-level resolution across all helminth life stages without requiring lethal sampling [20]. Furthermore, DNA metabarcoding allows for cost- and time-efficient high-throughput monitoring of helminth communities. Pre-processing techniques, such as flotation or sedimentation, which are often used to concentrate helminth biomass, generally do not add substantial value to species detection in metabarcoding. In this study, we employed metabarcoding to obtain our results, while a previous study on the same *Gazella gazella* population [34] used conventional methods. Our findings indicate notable differences between the two approaches, highlighting the impact of methodological choice on study outcomes. The combined use of metabarcoding and conventional methods appears to enhance diagnostic precision by leveraging the strengths of both approaches. Despite its advantages, metabarcoding is not without limitations, such as challenges in determining parasite viability and lifecycle stages. Combining metabarcoding with conventional methods could offer a more comprehensive assessment. For instance, while metabarcoding excels in identifying helminths from degraded samples, conventional methods could provide insights into parasite load and health impacts on gazelles by examining fresh specimens. This dual approach may also mitigate biases arising from the use of non-specific primers, a limitation noted in our study for certain taxa. Metabarcoding may excel in detecting a broader range of species, as it can identify specific gene sequences from even degraded samples, whereas conventional methods may provide more context on helminth viability through fresh sample analysis. Thus, integrating both methods could yield a more reliable and comprehensive strategy for parasite diagnostics, reducing the potential for biases associated with each individual approach.

The parasites identified in this study are expected to impact on the health and well-being of *Gazella gazella*. The application of next-generation sequencing (NGS) technologies in this study provides a robust framework for conservation genomics. By generating comprehensive profiles of parasite communities, metabarcoding aids in identifying threats to population health and supports targeted management interventions. For *Gazella gazella*, this molecular perspective could inform habitat restoration efforts and improve our understanding of interactions with sympatric species, including potential intermediate hosts or predators. The gastrointestinal system of these animals harbors a range of helminth parasites that cause both subclinical and clinical parasitism. Gastrointestinal parasites can lead to visible outcomes, such as reduced appetite and subsequent weight loss, in both wild and domestic grazing animals. With heavier parasitic loads, clinical symptoms including diarrhea, anemia, submandibular edema, and weight loss may occur. Lungworms, in particular, are known to cause respiratory symptoms affecting the lungs and bronchioles [93, 94]. These clinical symptoms negatively affect the animals’ health, leading to reduced productivity and potential economic losses. In livestock, gastrointestinal parasites can reduce feed intake and nutrient absorption, resulting in significant weight losses, slower growth rates, and decreased meat yield, causing an economic burden. Additionally, gastrointestinal parasites can compromise wool quality, impacting the wood yield important to the textile industry and increasing costs of producers. Studies have documented the serious clinical implications and importance of nematodes in animal health models [95–97]. Parasitic infections directly affect the welfare and overall health of wild species, highlighting the importance of comprehensive analyses of such parasites in wildlife populations like mountain gazelles for effective conservation and management. These studies also contribute to the ‘One Health’ perspective, encompassing environmental health factors, such as soil quality, water sources, and vegetation, that influence parasite life cycles, as well as the health of wildlife humans, and domestic animals. The identification of zoonotic parasites via metabarcoding underscores its significance within a ‘One Health’ framework, emphasizing the interrelation among wildlife, domestic animals, and human health. The capacity to identify zoonotic taxa through non-invasive methods highlights the significance of metabarcoding as an essential instrument for disease monitoring, especially in areas such as Hatay, where interactions between humans and wildlife are common because of overlapping grazing territories. Many parasites prevalent in wildlife have zoonotic potential, posing cross-species infection risks for humans and pets, which is increasingly significant concern [95].

The next-generation sequencing technology used in this study is a powerful tool that enables large-scale sequencing of genetic material and is widely applied to assess genetic similarities among different parasite species [98]. It is important to consider that a 100% genetic match between different parasite species may result from the conserved nature of certain regions, which are often evolutionarily preserved across species. Consequently, highly homologous genes or regions may appear identical among parasite species, leading to a 100% match. Primer length also significantly affects the species level identification capability of PCR-based studies. Short primers are more likely to bind to a broader region of the target area, increasing the likelihood of non-specific binding to homologous or similar regions across species. This can lead to unwanted amplification products and, thus, reduced PCR specificity. Moreover, the use of a single genetic region may be insufficient for reliable species differentiation, as certain genes or regions can be similar across closely related species due to genetic diversity. This genetic overlap may obscure species distinctions when relying solely on a single genetic marker. Moreover, detecting species previously unreported in Türkiye suggests that these species may not yet be catalogued for Türkiye in databases like NCBI (National Center for Biotechnology Information). This highlights the importance of comprehensive genetic databases to improve accuracy in identifying local biodiversity and highlights the potential for discovering undocumented species through expanded genetic analysis.

To enhance the utility of metabarcoding in helminth studies, future investigations should explore multi-gene approaches to improve taxonomic resolution and address primer specificity issues. Furthermore, the incorporation of eDNA sampling methods could facilitate more extensive ecological evaluations, including the identification of parasite occurrences in aquatic and terrestrial environments linked to gazelle habitats.

## Conclusion

Using DNA metabarcoding, we characterized the helminths present in a mountain gazelle population, establishing a baseline for future studies on parasite communities in mountain gazelles across broader geographic areas. The identification of eight helminth taxa, including several species previously unreported in Türkiye, highlights the value of advanced molecular techniques in uncovering parasite diversity. Furthermore, the alignment of the biological characteristics of the identified species with seasonal climatic parameters suggests that environmental conditions may play a significant role in shaping parasite dynamics. Overall, this research contributes not only to the conservation efforts for mountain gazelles but also emphasizes the need for ongoing surveillance and monitoring to address the ecological and zoonotic implications of helminth infections in wildlife. This study highlights the essential function of DNA metabarcoding in enhancing the taxonomic clarity and ecological insight of helminth communities in wildlife. It also showcases its potential as a fundamental element for incorporating molecular tools into conservation strategies, especially for endangered species such as *Gazella gazella*.

## Data Availability

No datasets were generated or analysed during the current study.
